# Dextrin-Based Adsorbents Synthesized via a Sustainable Approach for the Removal of Salicylic Acid from Water

**DOI:** 10.3390/nano13202805

**Published:** 2023-10-22

**Authors:** Claudio Cecone, Mario Iudici, Marco Ginepro, Marco Zanetti, Francesco Trotta, Pierangiola Bracco

**Affiliations:** 1 Department of Chemistry, Nis Interdepartmental Centre, University of Turin, Via P. Giuria 7, 10125 Turin, Italymarco.ginepro@unito.it (M.G.); marco.zanetti@unito.it (M.Z.); pierangiola.bracco@unito.it (P.B.); 2 INSTM Reference Centre, University of Turin, Via G. Quarello 15A, 10135 Turin, Italy; 3 ICxT Interdepartmental Centre, University of Turin, Via Lungo Dora Siena 100, 10153 Turin, Italy

**Keywords:** biobased polymers, dextrins, sustainable synthesis, adsorption, emerging contaminants

## Abstract

Pharmaceuticals such as salicylic acid are commonly detected in wastewater and surface waters, increasing concern for possible harmful effects on humans and the environment. Their difficult removal via conventional treatments raised the need for improved strategies, among which the development of bioderived adsorbents gained interest because of their sustainability and circularity. In this work, biobased cross-linked adsorbents, synthesized via a sustainable approach from starch derivatives, namely beta-cyclodextrins and maltodextrins, were at first characterized via FTIR-ATR, TGA, SEM, and elemental analysis, showing hydrophilic granular morphologies endowed with specific interaction sites and thermal stabilities higher than 300 °C. Subsequently, adsorption tests were carried out, aiming to assess the capabilities of such polymers on the removal of salicylic acid, as a case study, from water. Batch tests showed rapid kinetics of adsorption with a removal of salicylic acid higher than 90% and a maximum adsorption capacity of 17 mg/g. Accordingly, continuous fixed bed adsorption tests confirmed the good interaction between the polymers and salicylic acid, while the recycling of the adsorbents was successfully performed up to four cycles of use.

## 1. Introduction

Over the past two decades, encouraged by spreading the “Green Chemistry” concepts, much work has been conducted on developing sustainable and bio-derived polymers meant to be applied for various applications [1]. In this framework, the use of dextrins such as maltodextrins and cyclodextrins to produce polymer adsorbents for environmental applications has constantly increased [2,3,4,5,6,7,8,9]. Maltodextrins and cyclodextrins are D-glucose-water-soluble oligomers obtained from the hydrolysis of starch. The first display both α-(1,4) and α-(1,6) glycosidic domains and are characterized by a dextrose equivalent lower than 20, which represents the reducing equivalent of a carbohydrate against the same mass of glucose [10,11]. On the other hand, cyclodextrins are cyclic, truncated, cone-shaped molecules consisting of α-(1,4)-linked glucopyranose units, surrounding a slightly lipophilic inner cavity, enabling them to form inclusion complexes with target molecules [12,13]. The most common cyclodextrins available on the market are characterized by six, seven, and eight glucopyranose units and therefore defined as alpha-cyclodextrins, beta-cyclodextrins, and gamma-cyclodextrins, respectively [14,15,16]. Due to the water solubility of both maltodextrins and cyclodextrins, numerous studies have studied the possibility of cross-linking them by exploiting suitable cross-linkers such as carbodiimide, epichlorohydrin, glutaraldehyde, isocyanates, and poly-carboxylic acids to broaden their applications to those materials that require being insoluble in aqueous media [17,18,19,20,21,22]. However, many of these compounds exhibit toxicity and adverse environmental impacts. With the aim of identifying harmless cross-linkers, water-soluble diglycidyl ethers have been studied and reported, among which 1,4 butanediol diglycidyl (BDE) ether has revealed low-toxic and biocompatible features [23].

Xue et al. described the preparation of hydrogels obtained by cross-linking hyaluronic acid with BDE, showing that low cytotoxicity and suitable mechanical properties make them promising candidates for applications in regenerative medicine and tissue engineering [24]. Aerogels with high adsorbent ability were prepared by Liu et al. [25] from cellulose cross-linked with BDE, while a drug release system was developed by Li et al. [26] through the cross-linking of galactomannan with BDE. BDE cross-linked cyclodextrin/agar-based hydrogels as drug delivery systems were reported by Blanco-Fernandez et al. [27], while the use of BDE to cross-link cyclodextrins and maize-derived maltodextrins to obtain a suitable adsorbent has been the subject of recent studies by our group [28,29].

Active pharmaceutical ingredients, together with personal care products, pesticides, industrial additives, monomers, and plasticizers, are part of the so-called emerging contaminants or contaminants of concern because of their potential to cause undesirable effects on the environment or human health, their slow kinetics of biodegradation, and their resilience to conventional water treatment processes [30,31]. Even though they are compounds detected in the environment, they remain unregulated or are in the process of being regularized [32,33]. Pharmaceuticals like anti-inflammatory drugs, antibiotics, analgesics, hormones, β-blockers, blood lipid regulators, antiepileptics, and antidepressants are present in the environment at low but influencing concentrations. Although most of them are not highly persistent, their continuous addition to the environment from several sources causes many to be considered “pseudo-persistent” [34,35]. Nevertheless, due to the lack of experimental data, it is still not clear how these pollutants affect flora, fauna, the environment, and humans [36].

Widely employed in pharmaceutical, dermatological, and cosmetic formulations, salicylic acid (SA) and its derivatives are frequently detected in wastewater and surface waters at concentrations up to 10^2^ μg/L and up to 10^1^ μg/L, respectively, in Europe [37,38]. Concerning pharmaceuticals, SA is the precursor to acetylsalicylic acid, otherwise called aspirin, the most extensively consumed analgesic, antipyretic, and anti-inflammatory agent in the world [39]. Furthermore, thanks to its keratolytic, bacteriostatic, fungicidal, and photoprotective properties, SA is largely exploited for the topical treatment of, e.g., warts, localized hyperkeratosis, psoriasis, and comedonal acne; it is also included in skin ointments as a peeling agent; and it is due to the presence of an aromatic ring used in sunscreen preparations [40,41]. Eventually, SA has also been applied in households as a food preservative [41]. However, despite this large use, SA can cause acute and chronic toxicity known as salicylism, whose symptoms include nausea, vomiting, dizziness, confusion, delirium, stupor, psychosis, coma, and in the worst cases, even death [42,43]. For this reason, the removal of SA from water is a paramount necessity. Conventional treatment processes such as chlorination, filtration, and coagulation-flocculation are not effective in completely removing emerging contaminants from wastewater, surfaces, and drinking water [44,45,46]. For this reason, adsorption with activated carbons and oxidation by ozone are considered the present-day industry standard for this goal; nevertheless, these technologies are high-priced because of the cost related to the adsorbent in the first case and the costs associated with the process in the latter [47,48,49].

In the present work, bioderived neutral and cationic cross-linked polymers were first synthesized following a sustainable approach. Subsequently, as a result of previous studies in which this class of materials exhibited promising adsorption abilities towards both orange II and ciprofloxacin [28,29], the polymers were further tested as suitable adsorbents for the removal of salicylic acid as a case study for emerging pollutants decontaminating water.

## 2. Materials and Methods

Beta-cyclodextrins (βCD) and maltodextrins with a DE of 2 (Glucidex 2^®^, GLU2) were supplied by Roquette Freres (Lestrem, France). Approximately 1,4 butanediol diglycidyl ether (BDE) and 1,4-Diazabicyclo [2.2.2] octane (DABCO) were purchased from Sigma-Aldrich (Darmstadt, Germany). Βcd and GLU2 were desiccated at 75 °C before use.

### 2.1. Synthesis of Plain βCD-Based Polymer (βCD_BDE)

**Figure 1:**
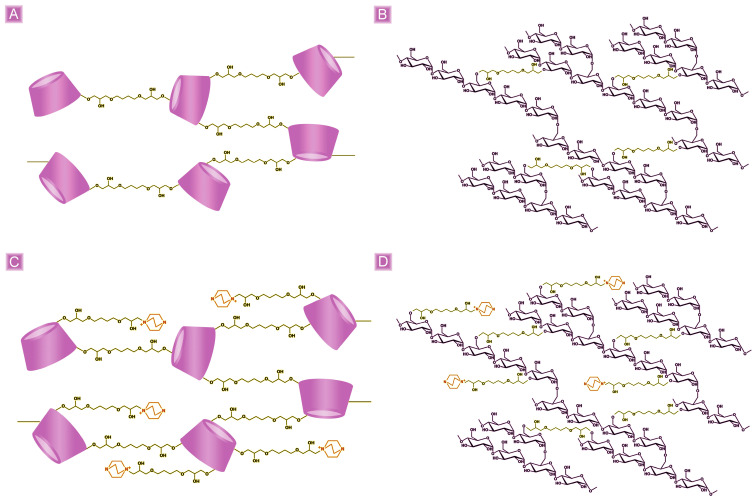
Schematic representation of (**A**) βCD_BDE, (**B**) GLU2_BDE, (**C**) βCD_BDE_Q^+^, and (**D**) GLU2_BDE_Q^+^.

In a typical procedure, the βCD_BDE polymer was synthesized by dissolving 5.00 g of βCD in 20 mL of a 0.2 M sodium hydroxide solution; thereafter, 6.50 mL of BDE was added while continuously stirring the solution, and the temperature was increased to 90 °C. The reaction was then allowed to proceed for 90 min, ultimately obtaining the product in the form of a monolith block. Subsequently, the product was crushed, allowing it to be recovered from the flask and purified with deionized water to remove any non-reacted reagents. At the end of the purification, the product was dried at 70 °C to a constant weight and finally ground with a mortar, obtaining a powder. The expected chemical structure of the synthesized polymer is reported in Figure 1A.

### 2.2. Synthesis of Plain GLU2-Based Polymer (GLU2_BDE)

In a typical procedure, the GLU2_BDE polymer was synthesized by dissolving 7.00 g of GLU2 in 20 mL of 0.2 M NaOH sodium hydroxide solution; thereafter, 1.50 mL of BDE was added while continuously stirring the solution, and the temperature was increased to 70 °C. The reaction was then allowed to proceed for 90 min, ultimately obtaining the product in the form of a monolith block. Subsequently, the product was crushed, allowing it to be recovered from the flask and purified with deionized water to remove any non-reacted reagents. At the end of the purification, the product was dried at 70 °C to a constant weight and finally ground with a mortar, obtaining a powder. The expected chemical structure of the synthesized polymer is reported in Figure 1B.

### 2.3. Synthesis of Cationic βCD-Based Polymer (βCD_BDE_Q^+^)

In a typical procedure, the βCD_BDE_Q^+^ polymer was synthesized by dissolving 7.50 g of βCD in 20 mL of 0.2 M sodium hydroxide solution; thereafter, 0.37 g of DABCO was added while continuously stirring the solution. Eventually, 4.85 mL of BDE was added, and the temperature was increased to 90 °C. The reaction was then allowed to proceed for 90 min, ultimately obtaining the product in the form of a monolith block. Subsequently, the product was crushed allowing it to be recovered from the flask and purified with deionized water, to remove any non-reacted reagents. At the end of the purification, the product was dried at 70 °C up to constant weight and finally ground with a mortar, obtaining a powder. The expected chemical structure of the synthesized polymer is reported in Figure 1C.

### 2.4. Synthesis of Cationic GLU2-Based Polymer (GLU2_BDE_Q^+^)

In a typical procedure, the GLU2_BDE_Q^+^ polymer was synthesized by dissolving 2.15 g of GLU2 in 20 mL of a 0.2 M sodium hydroxide solution; thereafter, 0.22 g of DABCO was added while continuously stirring the solution. Eventually, 1.56 mL of BDE was added, and the temperature was increased to 70 °C. The reaction was then allowed to proceed for 90 min, ultimately obtaining the product in the form of a monolith block. Subsequently, the product was crushed, allowing it to be recovered from the flask and purified with deionized water to remove any non-reacted reagents. At the end of the purification, the product was dried at 70 °C to a constant weight and finally ground with a mortar, obtaining a powder. The expected chemical structure of the synthesized polymer is reported in Figure 1D.

### 2.5. Characterization

Thermogravimetric analyses (TGA) were performed using a TA Instruments Q500 TGA (New Castle, DE, USA), from 50 °C to 700 °C, with a heating rate of 10 °C/min, under nitrogen flow.

The FTIR-ATR (Attenuated Total Reflection) characterization was performed using a Perkin Elmer Spectrum 100 FT-IR Spectrometer (Waltham, MA, USA) equipped with a Universal ATR Sampling Accessory. All spectra were acquired in the wavenumber range of 650–4000 cm^−1^, with a resolution of 4 cm^−1^ and 8 scans/spectrum, at room temperature.

Differential scanning calorimetry analyses (DSC) were performed using a TA Instruments Q200 DSC (New Castle, DE, USA), from 50 °C to 180 °C, with a heating rate of 10 °C/min, under nitrogen flow.

A Thermo Fisher FlashEA 1112 Series elemental analyzer (Waltham, MA, USA) was used to study the chemical composition of the samples.

A Tescan VEGA 3 (Brno, Czech Republic) scanning electron microscope (SEM) was used to study the morphology of the samples. The SEM images were acquired using secondary electrons and an 8 kV accelerating voltage. Before SEM characterization, the samples were ion-coated with 12 nm of gold using a Vac Coat DSR1 sputter coater (London, United Kingdom).

The pH of zero-point charge (pHZPC) of all polymers was measured following the pH drift method. A total of 20 mL of 0.01 M NaCl solutions were prepared by adjusting the pH within the range 2–10 (pH_i_) by adding HCl or NaOH. Afterwards, the solution pH was measured before (pH_i_) and after (pH_f_) 24 h of contact with 150 mg of the adsorbent under stirring. The intersection between the curves (pH_i_ versus pH_f_) obtained with and without the adsorbent represents the value of pH_ZPC_.

The swelling tests were carried out by adding 200 mg of the polymer obtained from each synthesis to 10 mL of distilled water. The samples were subsequently allowed to swell for 24 h. Afterwards, the liquid phase was removed via centrifugation, and the swelling percentage was calculated as follows:

(1) $$\%\;Swelling=\left(\frac{\;g_{\left(swelled\;polymer\right)}-g_{\left(dry\;polymer\right)}\;}{g_{\left(dry\;polymer\right)}}\right)*100$$

### 2.6. Salicylic Acid Adsorption Tests and HPLC-UV/Vis Detection

Batch adsorption tests were carried out starting from 25 mL of 1 and 10 mg/L SA water solutions at pH 7 and room temperature. A calibration curve in the range 0.1–1 mg/L was constructed for the test performed at 1 mg/L, while a calibration curve in the range 1–10 mg/L was constructed for the test performed at 10 mg/L. All the adsorption tests were performed in triplicate by adding 25 mg of the polymers to SA solutions. All the dispersions were continuously stirred with an orbital shaker and kept at room temperature. At fixed intervals, the concentration of SA was measured via HPLC-UV/Vis using a Dionex (Sunnyvale, CS, USA) instrument consisting of a P680 pump coupled with a UVD170U detector. Separation was achieved using a Kinetex^®^ C18 (150 × 4.6 mm, 5 μm). The mobile phase consisted of 50 mM phosphate buffer and acetonitrile in a ratio of 90:10 *v*/*v*. The mobile phase was filtered (0.45 μm nylon filter) and degassed before use. The quantification of SA was performed at 240 nm, with 0.1 mg/L as the detection limit. The run time for the assay was set at 5 min with 1 mL/min flow, while the retention time for SA was 3.5 min. Continuous fixed-bed adsorption tests were performed in a self-made apparatus composed of a 1 mL plastic syringe packed with 25 mg of adsorbent, which was left to swell for 24 h in deionized water before use. A 1 mg/mL SA solution reservoir, contained in a drip funnel, was connected to the top of the syringe, and the permeation flow, driven by gravity, was adjusted at 1.5 mL/min. The HPLC-UV/Vis detection was conducted as previously described for each 20 mL of solution permeated through the adsorbent.

## 3. Results and Discussion

### 3.1. Characterization of the Adsorbents

In addition to showing low toxicity and good biocompatibility, thanks to its water solubility, BDE allows for the avoidance of the use of organic solvents during synthetic procedures. It also gives excellent yields as a result of the atom economy of the epoxide ring-opening reactions. For each synthesis performed, the mass balance was calculated as the mass of the final product after purification and drying versus the theoretical mass, equal to the sum of the masses of GLU2, BDE, and DABCO when present. The mass balance was 80% for βCD_BDE, 76% for GLU2_BDE, 78% for βCD_BDE_Q^+^, and 82% for GLU2_BDE_Q^+^.

**Figure 2:**
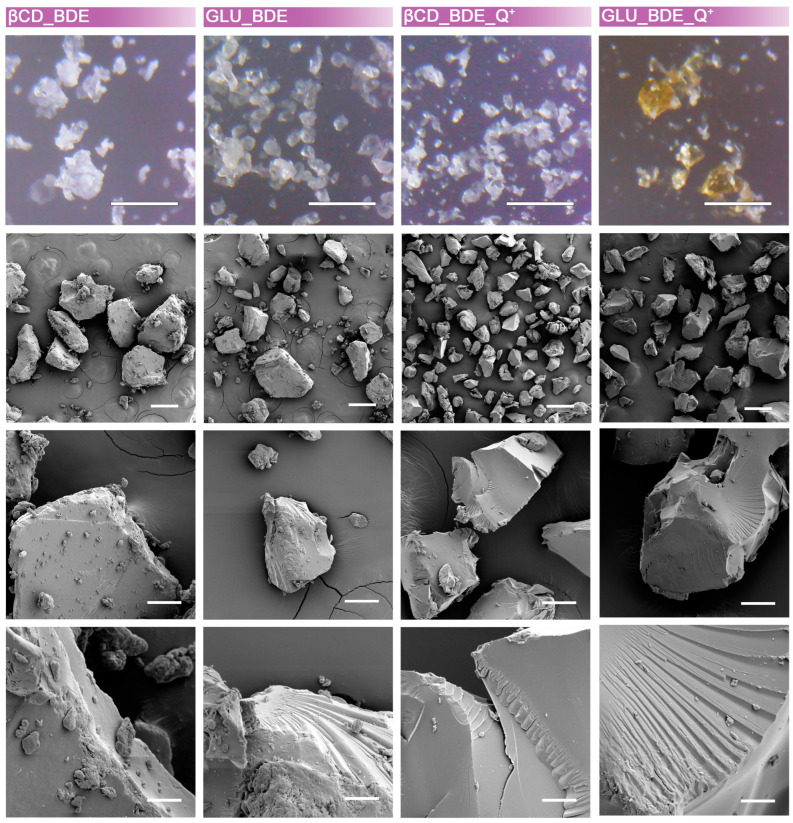
Microscope images and SEM characterization of polymer granules. Scale bars: 250 μm (first line); 200 μm (second line); 50 μm (third line); 10 μm (fourth line).

All the products were ground in a mortar to reduce the grain size, which eventually ranged, as evidenced by the SEM characterization reported in Figure 2, from tens to hundreds of microns. The grain size is a factor of great importance in both batch and continuous adsorption processes. Ideally, increasing the surface-to-volume ratio, therefore decreasing the grain size, would favor the contact between the adsorbent and the solution. However, small grain sizes could result in a difficult recovery of the absorbent in batch tests and a difficult permeation through the fixed bed in continuous adsorption tests. For this reason, an optimal size range should be evaluated depending on the characteristics of the adsorbent [50,51,52,53]. Eventually, no mesoporosity or macroporosity was detected.

The presence of crystallinity on BDE-linked dextrin-based polymers was investigated via XRD in a recent study by our group, where the materials were amorphous [28,29].

**Figure 3:**
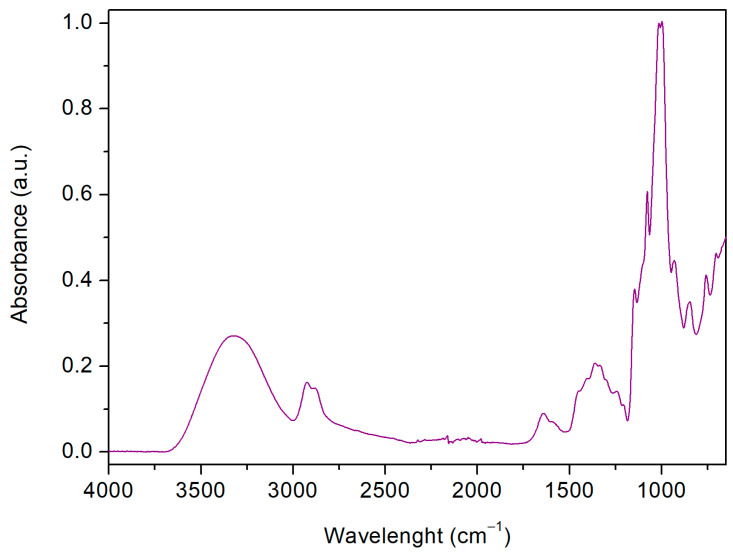
FTIR-ATR spectrum of GLU2_BDE_Q^+^.

Typical dextrin IR signals, as well as those belonging to the linker, were observed from the FTIR-ATR analysis of the polymers. Because of similarities between the FTIR-ATR analyses, only the spectrum of GLU2_BDE_Q^+^ is reported in Figure 3. A large band characterizing the spectral region between 3000 cm^−1^ and 3500 cm^−1^, associated with symmetric and anti-symmetric O–H stretching modes, was detected, together with the OH bending signal, occurring at 1645 cm^−1^. The C-O-C or C-O bond vibrations of both dextrins and BDE were visible in the region 1080–1000 cm^−1^. The bands observed at 2921 cm^−1^, and 2867 cm^−1^ are typical of C–H stretching modes, while in the regions 950–650 cm^−1^ and 1400–1150 cm^−1^ the C–H bonds, glucopyranose cycle vibrations, and C–H bond deformation belonging to primary and secondary hydroxyl groups were observed, respectively. Eventually, in the case of βCD_BDE_Q^+^ and GLU2_BDE_Q^+^ (Figure 3), the occurring of amino-mediated ring-opening reactions, resulting in quaternary ammonium functions, was detected as a shoulder at 1590 cm^−1^.

**Figure 4:**
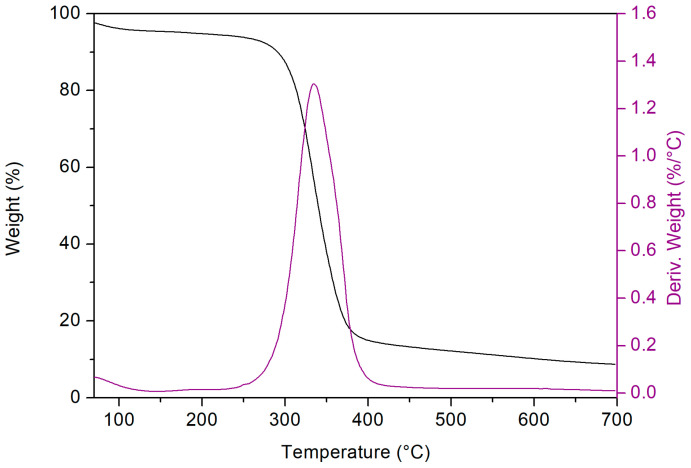
TGA and related DTGA of βCD_BDE_Q^+^.

The thermogravimetric profiles (because of similarities, only the TGA and DTGA of βCD_BDE_Q^+^ are reported in Figure 4) were characterized by a first weight loss phenomenon, occurring approximately up to 150 °C, which was related to the volatilization of the adsorbed water, comprising between 3% and 8%. Subsequently, a single-step decomposition process taking place between 250 °C and 450 °C was detected, with a maximum rate of decomposition, as evidenced by the derivative curves, centered roughly from 310 °C up to 370 °C. As a result of the polymer pyrolysis, a carbon residue ranging approximately from 10% to 15% of the initial weight and stable up to 700 °C was obtained.

Because of the hydrophilic characteristics of both βCD and GLU2, swelling tests were performed as the polymers were developed to be applied in water media. The ability of an adsorbent to swell in contact with water is an aspect that has to be taken into consideration. A swollen hydrophilic adsorbent may be able to let the solution permeate within the polymer matrix, allowing adsorption phenomena to occur not only at the surface. However, swollen grains might not retain their mechanical properties, making softened materials hard to handle and recover. Moreover, swelling phenomena might affect the permeation through the adsorbent in the case of continuous adsorption tests [50,51,52,53]. The swelling percentage resulted in 392 ± 59% for βCD_BDE, 344 ± 31% for GLU2_BDE, 685 ± 85% for βCD_BDE_Q^+^, and 634 ± 18% for GLU2_BDE_Q^+^. Inarguably, the presence of cationic sites deeply affected the swelling properties of the polymers. The addition of DABCO during the synthesis resulted in polymers with a swelling capacity 1.75-fold higher in the case of βCD-based synthesis and 1.84-fold higher in the case of GLU2-based synthesis, if compared to the same ones carried out without amine. This difference can be attributed to a higher polarity of the polymer network, responsible for increased interactions with the solvent. Furthermore, since the amine reacts with BDE epoxide rings, covalently bound to the polymer structure, it also affects the cross-linking density of the systems, allowing a less rigid network to be formed. Nitrogen atoms belonging to the amine-mediated reactive path and thus entering the polymer structure were confirmed via elemental analyses. βCD_BDE_Q^+^ and GLU2_BDE_Q^+^ showed a nitrogen content of 0.59 + 0.01 wt.% and 0.60 + 0.03 wt.%, respectively, whereas, as expected, no nitrogen was detected in βCD_BDE and GLU2_BDE.

### 3.2. SA Batch Adsorption Test

All polymers were first screened to assess their performance in adsorbing SA from water solutions. The first test was carried out by adding 25 mg of adsorbent to 25 mL of a 10 mg/L SA solution at pH 7. The adsorption of SA was followed at different time intervals of 15 min, 30 min, and 60 min, whereas the adsorbed amount was expressed as (i) adsorbed percentage (*Ads*_(%)_):

(2) $${Ads}_{\left(\%\right)}=\left(1-\frac{{Conc\;t}_{x}}{{Conc\;t}_{0}}\right)\times 100$$

and (ii) adsorption capacity, i.e., milligrams of SA adsorbed per gram of adsorbent (*Ads_mg/g_*):

(3) $${Ads}_{\left(mg/g\right)}=\left({Conc\;t}_{0}-{Conc\;t}_{x}\right)\times \frac{V}{m}$$

where *Conc t*_0_ (mg/L) represents the initial SA concentration, *Conc t_x_* (mg/L) is the concentration of SA after each time interval, *V* (L) is the volume of SA solution, and *m* (g) is the mass of the adsorbent used.

**Figure 5:**
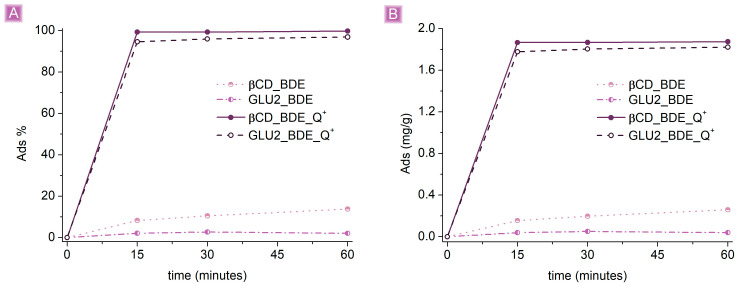
SA adsorption comparison (**A**) *Ads*_(%)_ vs. time (**B**) *Ads*_(*mg/g*)_ vs. time. A total of 125 mg of adsorbent was used in 25 mL of a 10 mg/L SA solution pH 7.

From the first screening (Figure 5), the effect of the presence of cationic sites appeared undeniable. The highest *Ads*_(%)_, nearly 100%, was observed for βCD_BDE_Q^+^, with an adsorption capacity, *Ads*_(*mg/g*)_, of 1.88 mg/g, followed by GLU2_BDE_Q+, with *Ads*_(%)_ corresponding to 96.9% and *Ads*_(*mg/g*)_ of 1.82 mg/g. On the other hand, βCD_BDE and GLU2_BDE showed *Ads*_(%)_ equal to 13.8% and 2.0%, respectively. This gap suggests an electrostatic nature of the interaction between the adsorbent and the drug. This hypothesis is supported by the pKa value of salicylic acid, equal to 2.97, which at pH 7 reflects the primary presence of negatively charged salicylate species capable of interacting with positively charged polymers, driven by electrostatic forces [54]. However, besides the predominant presence of electrostatic interactions, the slightly higher *Ads*_(%)_ displayed by the βCD-based adsorbents compared with GLU2-based syntheses reveal a more complex adsorption process composed of multiple phenomena.

**Figure 6:**
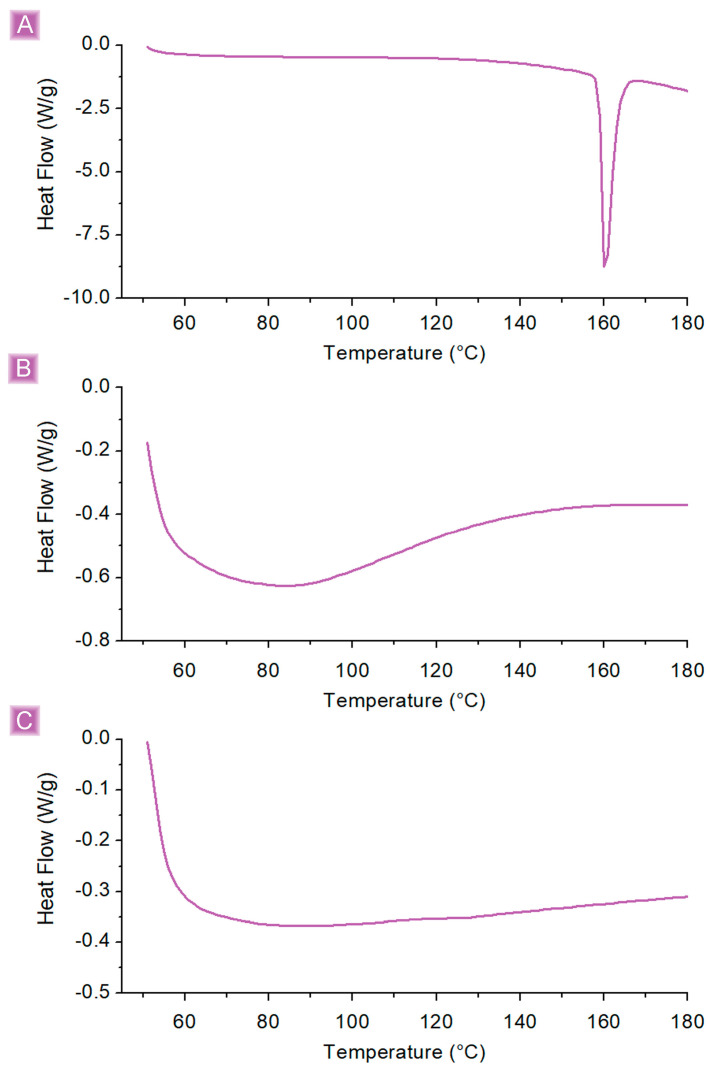
DSC analyses of (**A**) SA, (**B**) βCD_BDE_Q^+^, and (**C**) βCD_BDE_Q^+^ after the adsorption test.

Firstly, as widely reported in the literature from pristine βCD [55,56,57], the presence of βCD domains may endow the polymer matrix with the ability to form inclusion complexes with SA. However, the quantification of inclusion complex formation over electrostatic interaction is a questionable task for polymer systems. In the case of pristine βCD, a common strategy employs DSC, where the absence of the drug melting signal is associated with a successful encapsulation, the drug being present as a molecular inclusion complex and thus not capable of crystallizing [58,59]. Nevertheless, as reported in Figure 6, although the absence of the SA melting signal (160.5 °C) would point in favor of host-guest formation, the reason could also be related to the relatively low amount of adsorbed drug, giving phenomena of intensity below the sensibility of the instrument. Along with the formation of the inclusion complex, a further possible interaction could be related to the cross-linking density itself. A general feature of cross-linked networks, such as those synthesized during this work, is the ability to prevent target molecules permeated within the polymer matrix as a consequence of diffusive transfer and swelling phenomena from escaping the adsorbent granules, thanks to steric trapping, probably the scenario observed from GLU2_BDE *Ads*_(%)_. However, as suggested by the almost negligible *Ads*_(%)_, this last phenomenon is reasonably of minor relevance if compared to the inclusion of complex formation and the leading electrostatic interaction.

Being the best-performing adsorbent, βCD_BDE_Q^+^ was selected for the following part of the work, which focused on studying the kinetics and influence on the adsorption performance of parameters such as the amount of adsorbent, the concentration of SA solution, the pH, and the presence of other chemical species in solution.

**Figure 7:**
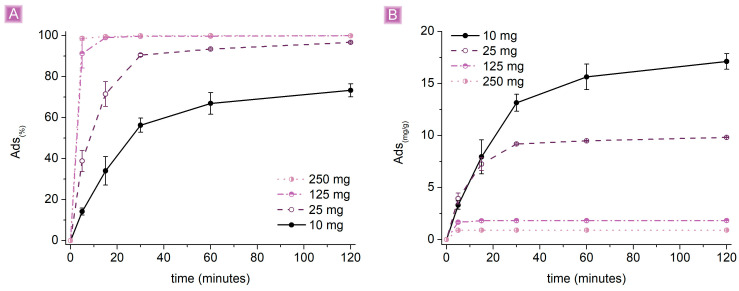
Adsorption performances (25 mL of 10 mg/L SA solution) as a result of the amount of adsorbent (**A**) *Ads*_(%)_ vs. time (**B**) *Ads*_(*mg/g*)_ vs. time.

Figure 7 reports the adsorption performances of βCD_BDE_Q^+^, observed by changing the amount of adsorbent, while keeping the constant SA concentration at 10 mg/L, pH 7, and the volume of solution at 25 mL.

An increase in the amount of adsorbent resulted in higher *Ads*_(%),_ associated with lower *Ads*_(*mg/g*)_ values. This trend can be explained by considering the electrostatic nature behind the predominant adsorption mechanism and the ratio between the active sites belonging to the adsorbent and the moles of drug present in the solution. Hypothesizing the homogeneous activity of all sites, they will interact with SA until their saturation. If the number of active sites is higher than the moles of SA, total adsorption will be observed; alternatively, a residual amount of SA will remain in the solution and subsequently be detected. When the number of active sites exceeds the moles of SA, a complete removal will occur, correlated with a proportional decrease in *Ads*_(*mg/g*)_ with the increase in the excess of active sites. Reasonably, in the presence of electrostatic interactions only, the higher *Ads*_(*mg/g*)_ values can be reached by saturating all active sites. However, as described above, the presence of multiple adsorption mechanisms and the presence of a swellable polymer matrix can affect the final performance. Considering 120 min of contact time, total *Ads*_(%)_ were obtained using both 250 mg, 125 mg, and 25 mg of adsorbent, while in the case of 10 mg, the value stood at 73.3 ± 3.2%. Also, with higher amounts of βCD_BDE_Q^+^, the adsorption reached its plateau faster because of the increasing number of active sites due to the increasing amount of adsorbent. By using 250 mg and 125 mg, the plateau is reached in a few minutes, probably because the sites exposed on the surface of the polymer granules are already sufficient to achieve complete removal of SA. Conversely, using 25 mg or even 10 mg of adsorbent, the removal occurs more slowly and reasonably due to sites accessible only after swelling of the granules and permeation of the SA solution within the polymer matrix. However, the higher *Ads*_(*mg/g*)_ were observed using 10 mg of βCD_BDE_Q^+^, resulting in 17.12 ± 0.76 mg/g, showing a good efficiency of all site types. 

**Figure 8:**
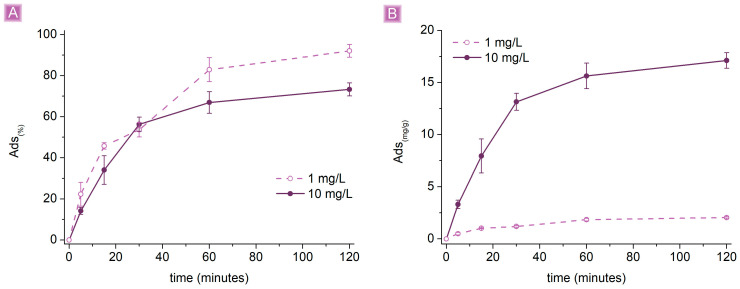
Adsorption performances (10 mg of βCD_BDE_Q^+^ in 25 mL SA solution) as a result of the concentration of SA (**A**) *Ads*_(%)_ vs. time (**B**) *Ads*_(*mg/g*)_ vs. time.

Keeping the amount of adsorbent constant at 10 mg, pH 7, and decreasing the SA concentration from 10 mg/L to 1 mg/L, the results obtained from the adsorption tests were consistent with the hypothesized behavior (Figure 8). A higher concentration of SA in the solution saturated the active sites of the adsorbent without achieving complete removal. On the other hand, the saturation of the active sites corresponded also to higher *Ads*_(*mg/g*)_; 1 mg/L SA solution gave an *Ads*_(*mg/g*)_ of 2.05 ± 0.07 mg/g, while an *Ads*_(*mg/g*)_ of 17.12 ± 0.75 mg/g was observed for the 10 mg/L SA solution, describing, in this specific case, a change of roughly one magnitude order also for the adsorption performance.

Pseudo-first order and pseudo-second order models were adopted for the analysis of kinetic data, at 298 K (Table 1), as follows:

Pseudo-first order

(4) $$\frac{dq_{t}}{dt}=k_{1}\left(q_{e}-q_{t}\right)$$

(5) $$\ln\left(q_{e}-q_{t}\right)=lnq_{e}-k_{1}t$$

Pseudo-second order

(6) $$\frac{dq_{t}}{dt}={k_{2}(q_{e}-q_{t})}^{2}$$

(7) $$\frac{t}{q_{e}}=\frac{1}{k_{2}q_{e}^{2}}+\frac{1}{q_{e}}$$

where *q_t_* and *q_e_* (mg/g) correspond to the adsorption capacities towards SA at contact time *t* (min) and equilibrium time, respectively. Whereas *k*_1_ (L/min) and *k*_2_ (g/mg·min) represent the adsorption rate constants of the pseudo-first-order and pseudo-second order equation, as reported in Table 1, a good fit with both pseudo-first-order and pseudo-second-order rate equations was observed for the adsorption of SA on βCD_BDE_Q^+^.

A comparison of the highest reported *Ads*_(*mg/g*)_ toward SA of various types of adsorbents is reported in Table 2. The performance displayed by βCD_BDE_Q^+^ resulted in the lowest among the studies considered. However, the conditions used in this work, namely low SA concentration and low adsorbent quantities, are the closest conditions likely to be found and expected for the decontamination of a real-world scenario, which is the aim of this study.

**Figure 9:**
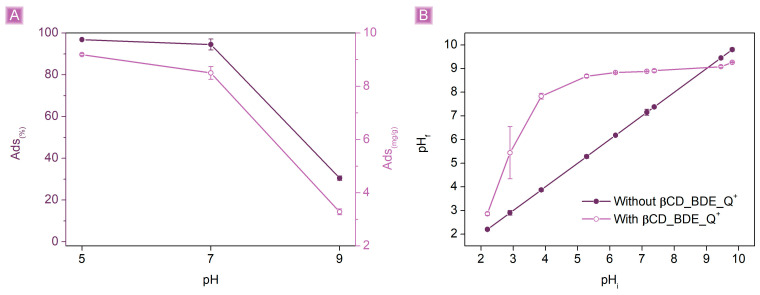
(**A**) Adsorption performances (25 mg of βCD_BDE_Q^+^ in 25 mL 10 mg/L SA solution, 120 min contact time) as a result of the pH and (**B**) pH_ZPC_ of βCD_BDE_Q^+^.

The effects of pH were subsequently evaluated on the adsorption performances of βCD_BDE_Q^+^ towards SA (Figure 9A). The pH was adjusted from 5 to 9 by adding aliquots of HCl 0.1 M or NaOH 0.1 M while keeping constant the amount of adsorbent at 25 mg, the volume, and the concentration of SA solution at 25 mL and 10 mg/L, respectively. The *Ads*_(%)_ were unaffected at acidic pH, and the SA removal remained nearly complete (96.9 ± 0.3%). Nevertheless, at high pH values, the performances dropped dramatically, down to 30.5 ± 1.0%, indicating a pH dependency on the adsorption process. As discussed above, SA is mostly in its salicylate form, above a pH of approximately 3; therefore, since the tests were conducted starting from pH 5, dissociation equilibria are not expected to occur on SA molecules, and the mechanisms underlying the observed behavior may be related to the adsorbent. A commonly used method to study the charge density of an adsorbent as a function of the pH of the medium in which the latter is dispersed is represented by the identification of the so-called pH of zero charges (pH_ZPC_). pH_ZPC_ reflects the condition in which equilibrium is reached between the number of positive and negative charges on the surface of an adsorbent, i.e., when the initial pH of the solution in which the adsorbent is tested is maintained after the dispersion is formed. As reported in Figure 9B, the pH_ZPC_ of βCD_BDE_Q^+^ resulted in 9.05, indicating that the polymer granules were positively charged below a pH of 9.05 and negatively charged for higher pH values. These results are consistent with the decrease in performances observed when the test was performed at basic pH; in this condition, the positive amino charges, the main driving force for the electrostatic interaction with SA, were mostly deprotonated and therefore no longer active. Additionally, a further aspect affecting the decrease in performances at basic pH that needs to be considered is the presence, at increasing pH values, of a higher concentration of hydroxyl species. The presence of these anions generates competition for the interaction with the cationic sites of the adsorbent, resulting in lower adsorption performances at higher pH values.

**Figure 10:**
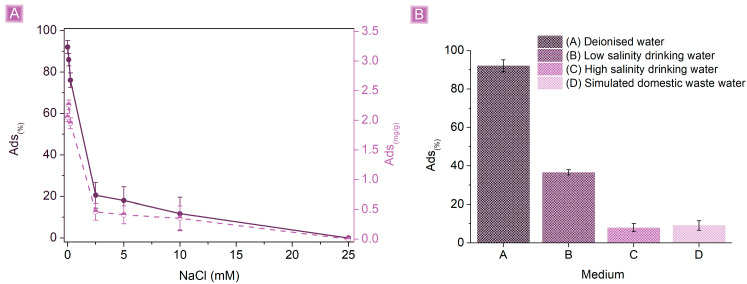
(**A**) Effect of NaCl concentration on SA adsorption and (**B**) SA adsorption performances in different aqueous media. Tests were performed using 10 mg of adsorbent in 25 mL of a 1 mg/L SA solution.

Further evaluation on the capability of βCD_BDE_Q^+^ to remove SA in non-ideal conditions was carried out by performing adsorption tests in (i) SA solutions containing increasing amounts of NaCl (from 0.1 mM to 25 mM), (ii) simulated SA-contaminated drinking water at high and low salinity, and (iii) simulated domestic wastewater (composition reported in Table 3) [66]. As reported in Figure 10, the *Ads*_(%)_ of βCD_BDE_Q^+^ are affected by the presence of the solution salinity, as a confirmation of electrostatic interactions as the predominant phenomena taking place in the adsorption mechanisms. The effect of NaCl appeared more evident as its concentration increased. Up to a NaCl concentration of 0.25 mM, corresponding approximately to 0.15 mg/L, the *Ads*_(%)_ resulted higher than 75%, decreasing to 20.5 ± 6.1% for NaCl solutions of 2.5 mM (1.5 mg/L) and dropping down to zero for NaCl concentrations of 25 mM (15 mg/L). The increasing concentration of NaCl, associated with higher amounts of chloride anions present in the solution, resulted in a competition for the cationic sites of the adsorbent and a decrease in adsorbing performances. The results of the tests performed on drinking waters contaminated with 1 mg/L of SA confirmed that the presence of salts, and mostly anions, has a strong influence on the decrease in SA removal. The *Ads*_(%)_ resulted in 36.6 ± 1.4% and 7.9 ± 2.1% in the cases of low and high salinity, respectively. In this regard, inorganic anions, being characterized by greater mobility and smaller dimensions compared to SA, allow kinetically favored and more stable interactions with the adsorbent, hindering the interactions of SA or displacing the already adsorbed SA molecules. Eventually, in the case of simulated domestic wastewater, the presence of a large amount of salts and organics (COD of 300 mg/L) resulted in the removal of SA being less than 10%. The observed results indicate that the potential application of βCD_BDE_Q^+^ as a suitable adsorbent would be limited to the decontamination of drinking waters characterized by low salinity. 

### 3.3. SA Fixed Bed Adsorption Test

**Figure 11:**
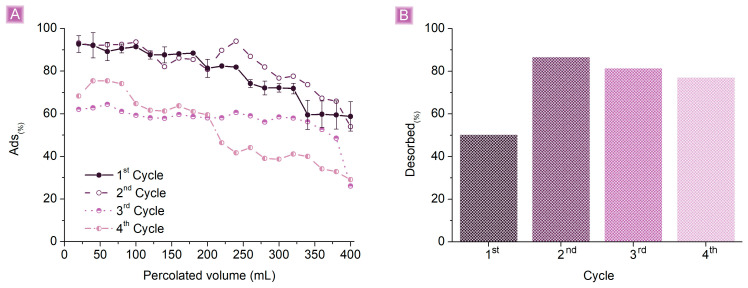
(**A**) SA column adsorption tests using 20 mg of adsorbent and 1 mg/L SA solution. (**B**) Regeneration cycles of βCD_BDE_Q+.

A second set of tests was performed, aiming to study more in detail the potential applicability of βCD_BDE_Q^+^ in continuous scenarios. A plastic column was filled with 20 mg of βCD_BDE_Q^+^ to carry out continuous fixed bed adsorption tests of a 1 mg/mL SA solution. The solution was allowed to permeate through the adsorbent at a flow rate of 1.5 mL/min, and SA concentration was monitored for each 20 mL of solution permeated. The *Ads*_(%)_ as a function of the permeated volume (Figure 11) resulted in approximately 90% for the first 100 mL and higher than 80% up to 240 mL of solution treated. Afterwards, the performance of the adsorbent decreased to approximately 70% at 320 mL, until approximately 60% at the end of the test, which was stopped at 400 mL of permeated solution. Considering the volume, the concentration of SA, and the adsorbent amount, the *Ads*_(*mg/g*)_ were equal to 16.17 mg/g. The possibility of reusing the adsorbent was investigated by washing βCD_BDE_Q^+^ after the test without displacing it from the column with 50 mL of a 0.1 M NaCl water solution to remove the SA molecules electrostatically bound to the adsorbent and restore the cationic sites. The displacement of SA from βCD_BDE_Q^+^ and thus the regeneration of the adsorbent were monitored by quantifying the SA present in the NaCl solution after contact with βCD_BDE_Q^+^, while the retention of the adsorbing performances was assessed by repeating the previously described SA continuous adsorption test after removing the NaCl excess from the adsorbent with 20 mL of deionized water. Following the same procedure, βCD_BDE_Q^+^ was tested for up to four reuse cycles. As reported in Figure 11A, the performances obtained in the first cycle were also maintained in the second cycle of use. However, from the third cycle, the *Ads*_(%)_ decreased by about 30%, even for the early stages of the test, starting with values comprised between 60% and 70% and ending with *Ads*_(%)_ in the range of 25–30%. The decreasing performances in the removal of SA are consistent with the quantification of SA displaced from βCD_BDE_Q+, which resulted, especially in the first cycle, lower than the total. This aspect reflects the non-optimal ability of the NaCl 0.1 M solution to displace SA from the adsorbent as a result of its insufficient ionic strength and the presence of SA molecules retained as inclusion complexes and thus not bound via electrostatic interactions. In this regard, better results could reasonably be achieved by testing more concentrated saline solutions or by adding aliquots of ethanol to recover SA from the host-guest interaction.

**Figure 12:**
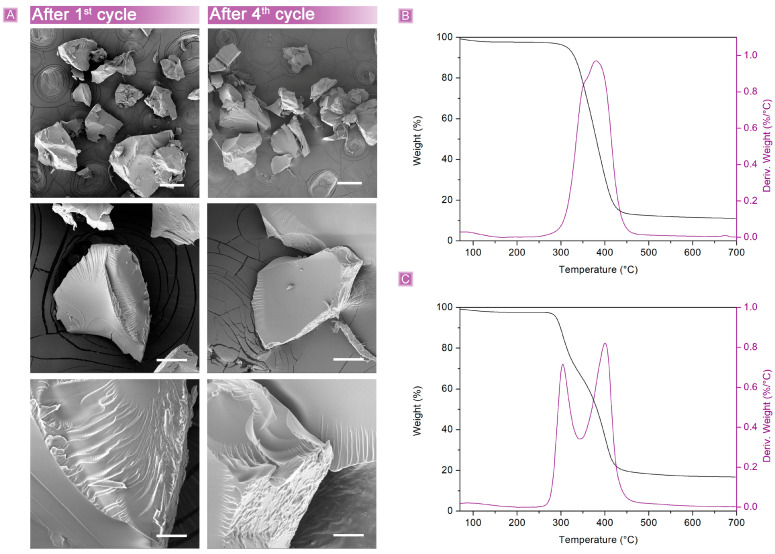
βCD_BDE_Q^+^ (**A**) SEM characterization and TGA after (**B**) the first and (**C**) fourth cycles of use in fixed bed continuous adsorption tests. Scale bars: 200 μm (first line); 50 μm (second line); 10 μm (third line).

However, the dried polymer granules appeared not to be damaged after both the first and fourth adsorption cycles (Figure 12A). The size distribution, as well as the surface features, were still consistent with those before use, suggesting that degradation phenomena, if present, were of negligible extent. Accordingly, the TGA showed a similar profile after the first cycle, with a decrease of roughly 20 °C in the T_onset_ (Figure 12B). However, instead of a single step, a two-step degradation profile was observed in the adsorbent recovered after the fourth cycle, associated with a decrease in the degradation T_onset_ of approximately 60 °C (Figure 12C). A possible explanation can be attributed to the mobility of polar polymer chains and rearrangements of the network as a result of the prolonged swollen conditions, as well as to the changes in salinity and the retention of a larger quantity of SA and salts after the drying process.

## 4. Conclusions

Four different cross-linked polymers, obtained from starch derivatives, were screened as suitable adsorbents for the removal of salicylic acid (SA) from water. Beta-cyclodextrins (βCD) and maltodextrins with dextrose equivalent of 2 (GLU2) were employed as the building blocks, whereas 1,4 butanediol diglycidyl ether (BDE) was chosen as the cross-linker, allowing the syntheses to be carried out in water media and thanks to the excellent atom economy of the epoxide ring-opening reaction taking place during the sol-gel polymerization procedure. Alongside the polymers obtained by cross-linking each building block with BDE, namely βCD_BDE and GLU2_BDE, the addition of the amine 1,4-diazabicyclo [2.2.2] octane, during the synthetic step, allowed to obtain positively charged products, βCD_BDE_Q^+^ and GLU2_BDE_Q^+^, respectively. The polymers were morphologically characterized via SEM, showing granules with smooth surfaces and dimensions ranging from tens to hundreds of microns. Their thermal stability was higher than 300 °C, according to the T_onset_ extrapolated from the corresponding TGA. Eventually, the presence of cationic functionalities was demonstrated through both elemental analyses and pH of zero charge measurements. From the first adsorption test, aimed at screening for the best-performing adsorbent, the presence of βCD domains associated with host-guest inclusion complex formation and mostly the presence of cationic functionalities related to the generation of electrostatic interaction with SA appeared pivotal in affecting the removal of SA. βCD_BDE_Q^+^ was the most performing system, showing a removal rate higher than 90% with an adsorption capacity of 2 mg/g from 1 mg/L of SA solution, employing 0.4 mg of adsorbent per mL of SA solution. A removal efficiency of 73% with an adsorption capacity of 17 mg/g was observed instead for a 10 mg/mL SA solution using 0.4 mg of adsorbent per mL of SA solution. SA dissociation equilibria, pH of zero-point charge, and competition with hydroxy species revealed an optimal pH range of 5 to 7 to carry out the adsorptions, whereas the presence in solution of salts and organics appeared detrimental to the removal of SA, suggesting how these adsorbents would be most suited for the decontamination of low salinity waters. Continuous fixed-bed adsorption tests were carried out as a proof of concept for continuous water treatment applications. Interestingly, 20 mg of βCD_BDE_Q^+^ allowed a removal higher than 90% for the first 100 mL, which gradually decreased to 60% until the end of the test and stopped at 400 mL of SA. A total of 1 mg/L solution permeated, corresponding to 16 mg/g adsorption capacity. The recycling of the adsorbent was also evaluated for up to four cycles of use. Performances dropped by about 30% on cycles three and four, due to incomplete displacement of the preciously adsorbed SA during the regeneration phase. Overall, the simplicity and sustainability of the synthetic procedures, the good adsorption performances displayed in both batch and continuous tests, and the good stability over time and cycle of use make this class of adsorbents promising candidates for further application in water remediation studies.

## Figures and Tables

**Table 1:** Correlative parameters of adsorption kinetics for the SA-βCD_BDE_Q^+^ system at 298 K.

Conc t_0_ (mg/L)	Ads Exp (mg/g)	Pseudo First Order			Pseudo Second Order		
		Ads Cal (mg/g)	k_1_	R^2^	Ads Cal (mg/g)	k_2_	R^2^
1	2.05	2.00	3.05 × 10^−4^	0.973	2.43	2.15 × 10^−2^	0.989
10	17.12	16.49	3.45 × 10^−4^	0.989	20.63	2.63 × 10^−3^	0.993

**Table 2:** Comparison of the highest reported *Ads*_(*mg/g*)_ toward SA of various adsorbents.

Adsorbent	*Ads*_(*mg/g*)_	SA (mg/L)	m (g/L)	T (°C)	t(h)	pH	Ref.
Cross-linked PS	396.8	700	4.0	10	24	2.6	[60]
Modified SiO_2_/Al_2_O_3_	256.1	1000	1.6	25	0.5	3.5	[61]
Cross-linked PMADETA/PDVB	238.3	1000	2.0	45	8	\	[62]
Barely straw biochar	210.5	250	0.5	45	11	3.0	[63]
Douglas fir biochar	108.8	>350	2.0	45	0.03	5.0	[64]
Pine wood biochar	22.7	>400	4.0	45	16	3.0	[65]
Dextrin-based polymer	17.1	10	0.4	25	2	7.0	This work

**Table 3:** Composition of simulated SA-contaminated drinking water at high and low salinity and simulated domestic wastewater [66].

Composition	Drinking Water		Simulated Domestic Wastewater
	High Salinity	Low Salinity	
SA (mg/L)	1	1	1
Ca^+2^ (mg/L)	400	9.5	-
Na^+^ (mg/L)	50	1.2	-
Mg^+2^ (mg/L)	25	2.6	-
K^+^ (mg/L)	49	0.53	-
SO_4_^−2^ (mg/L)	4.7	4.4	-
Cl^−^ (mg/L)	16	0.25	-
NO_3_^−^ (mg/L)	3.5	-	-
F^−^ (mg/L)	1	-	-
Fixed residue (mg/L)	1323	45.9	-
pH	6.2	7.2	7.1
Milk powder (mg/L)	-	-	150
Starch (mg/L)	-	-	80
Sodium acetate (mg/L)	-	-	103
Yeast (mg/L)	-	-	24
NH_4_Cl (mg/L)	-	-	21.7
Urea (mg/L)	-	-	12.8
KH_2_PO_4_ (mg/L)	-	-	13.2
NaHCO_3_ (mg/L)	-	-	600

## Data Availability

All data generated or analyzed during this study are included in this article.
